# Porcine Gasdermin D Is a Substrate of Caspase-1 and an Executioner of Pyroptosis

**DOI:** 10.3389/fimmu.2022.828911

**Published:** 2022-03-14

**Authors:** Yueyang Song, Jiameng Song, Meng Wang, Junwei Wang, Bo Ma, Wenlong Zhang

**Affiliations:** ^1^ College of Veterinary Medicine, Northeast Agricultural University, Harbin, China; ^2^ Northeastern Science Inspection Station, China Ministry of Agriculture Key Laboratory of Animal Pathogen Biology, Harbin, China

**Keywords:** porcine GSDMD, porcine caspase-1, cleavage, substrate, pyroptosis

## Abstract

Gasdermin (GSDM) family proteins were recently identified as the executioner of pyroptosis. The mechanism of pyroptosis mediated by gasdermin D (GSDMD) (a member of GSDM family) in humans and mice is well understood. In pyroptosis, mouse and human GSDMDs are cleaved by activated proinflammatory caspases (caspase-1, 4, 5, or 11) to produce anamino-terminal domain (GSDMD-NT) and a carboxyl-terminal domain (GSDMD-CT). The GSDMD-NT drives cell membrane rupture, which leads to the pyroptotic death of the cells. The expression of porcine GSDMD (pGSDMD) has recently been determined, but the activation and regulation mechanism of pGSDMD and its ability to mediate pyroptosis are largely unknown. In the present study, the activation of porcine caspase-1 (pcaspase-1) and cleavage of pGSDMD occurred in the duodenum and jejunum of a piglet challenged with enterotoxigenic *Escherichia coli* were first determined. Then the capability of pcaspase-1 to cleave pGSDMD was determined in a cell-free system and in human embryonic kidney cells. The pGSDMD cleavage by pcaspase-1 occurred after the pGSDMD molecule’s _276_Phenylalanine-Glutamine-Serine-Aspartic acid_279_ motif. The pGSDMD-NT generated from the pGSDMD cleavage by pcaspase-1 showed the ability to drive cell membrane rupture in eukaryotic cells. When expressed in *E. coli* competent cells, pGSDMD-NT showed bactericidal activity. These results suggest that pGSDMD is a substate of pcaspase-1 and an executioner of pyroptosis. Our work sheds light on pGSDMD’s activation mechanisms and functions.

## Introduction

Pyroptosis is a type of programmed cell death (1) that is characterized by cell swelling, breakdown of the plasma membrane, release of cytoplasmic contents, and vigorous inflammatory immune responses. Pyroptosis has been proven to be closely related to various human diseases, including infectious (2–4) and non-infectious diseases (5–7).

Members of gasdermin (GSDM) family have recently been proven as the executioners of pyroptosis (8–10). Gasdermin A (GSDMA), GSDMB, GSDMC, GSDMD, GSDME (also known as deafness, autosomal dominant 5, DFNA5), and DFNB59 are the six GSDMs that have been identified in humans (11, 12). GSDMD is the most widely studied GSDM protein in humans and mice. The cleavage of GSDMD by human and mouse caspase-1 or by human caspase-4/5/8 and mouse caspase-8/11 is the critical event of pyroptosis (8, 13, 14). The amino-terminal domain of GSDMD (GSDMD-NT), one of the caspase cleavage products, oligomerizes and forms pores in the plasma membrane, leading to pyroptotic cell death and thereby causes the release of proinflammatory cell components (15, 16).

In contrast with the extensive research on GSDM-mediated pyroptosis in humans and mice, research on the underlying mechanisms of cell pyroptosis in other species, such as pigs, is limited. An early study determined that *Salmonella typhimurium* infection increased caspase-1 expression to induce pyroptosis in porcine mesenteric lymph nodes (17). A recent study found that pathogenic *Escherichia coli* (*E. coli*) induced the upregulation of mRNA levels of NOD-like receptor (NLR) family, pyrin domain-containing protein 3 (NLRP3), apoptosis-associated speck-like protein containing CARD (ASC), caspase-1, IL-1β, IL-18, and GSDMD in infected pigs (18). Another recent work reported that classical swine fever virus (CSFV) infection activates caspase-1 and promotes GSDMD cleavage to produce GSDMD-NT in the peripheral immune organs of pigs and porcine peripheral blood monocytes (19, 20). Transmissible gastroenteritis virus (TGEV) has been reported to cause caspase-1 activation and GSDMD cleavage in porcine intestinal epithelial cells (21). These studies seemingly provide an explanation for the mechanism of cell pyroptosis in pigs. However, first, these studies enrolled human or mice GSDMD-specific antibodies as research tools, which creates doubt on whether their findings accurately reflect the cleavage of porcine GSDMD (pGSDMD). Second, if the GSDMD cleavage, which had been observed in these studies, indeed occurred, then whether a direct relationship exists between caspase-1 activation and GSDMD cleavage is unclear. Third, whether the cleavage products of pGSDMD can cause pyroptotic cell death remains unclear. The pyroptosis in pig thus deserves a separate research.

In the current study, we used self-prepared pGSDMD and porcine caspase-1 (pcaspase-1)-specific antibody and found that pcaspase-1 activation and pGSDMD cleavage occurred in the small intestine of a piglet challenged with enterotoxigenic *E. coli*(ETEC). We then found that pcaspase-1 can cleave pGSDMD between Aspartic acid (D)279 and Glycine (G)280. The pGSDMD-NT demonstrates the ability to cause pyroptosis in different cells lines and bactericidal activity. Our work sheds light on pGSDMD’s activation mechanisms and functions.

## Materials and Methods

### Plasmids and Antibodies

First, pUC57-pcaspase-1, a plasmid that contain the full-length porcine caspase-1 gene (GenBank Accession No.AB027296), was synthesized and constructed by GenScript (Nanjing, China). pET-30a(+)-His-pGSDMD, a plasmid that contains the full-length pGSDMD gene, was previously constructed in our laboratory (22).

The eukaryotic and prokaryotic expression plasmids that contain the nucleic acids encoding full-length/truncated pGSDMD or pcaspase-1 were constructed *via* standard PCR cloning strategy using the primers listed in **Table 1**. A prokaryotic expression plasmid that contains the nucleic acid encoding a mutant of pGSDMD was constructed from the pET-30a(+)-His-pGSDMD plasmid using the primers listed in **Table 1** and QuickMutation™ Site-Directed Mutagenesis Kit(Beyotime, Beijing, China) according to the manufacturer’s instructions. All recombinant plasmids were verified by DNA sequencing. The resulting constructions mentioned above are listed in **Table 2**.

**Table 1:** Primers used for plasmid construction.

Name of plasmids	Nucleotide of primer (5′ to 3′)	Restrict sites
pET-30a(+)-pcaspase-1 p20	GAAGATCTGGACGACGACGACAAGAACCCAGTTAAGCCTG	*Bgl* II
	CCGGAATTCTTAATCTTTTATCCATACG	*Eco*R I
pET-30a(+)-His-pcaspase-1	CCGGAATTCATGGCCGATAAGGTGC	*Eco*R I
	CGGCTCGAGTTAATGTCCTGGGAAG	*Xho* I
pET-30a(+)-pcaspase-1-His	CGCCATATGGCCGATAAGGTGCTG	*Nde* I
	CGGCTCGAGATGTCCTGGGAAGAG	*Xho* I
pET-30a(+)-Δpcaspase-1-His	CGCCATATGAACCCAGTTAAGCCTG	*Nde* I
	CGGCTCGAGATGTCCTGGGAAGAG	*Xho* I
pcDNA3.1(+)-pcaspase-1 p20	CCGGAATTCACCATGGCTAACCCAGTTAAGCC	*Eco*R I
	CGGCTCGAGTTAATCTTTTATCCATACG	*Xho* I
pEGFP-C1-pGSDMD	CCGGAATTCGATGGCATCAGCCTTTGAG	*Eco*R I
	GCTCTAGACTAGCAGAGCTGGCTGAG	*Xba* I
pET-30a(+)-His-pGSDMD-NT	CCGGAATTCATGGCATCAGCCTTTGAG	*Eco*R I
	ACGCGTCGACTTAGTCTGACTGGAACTTCAG	*Sal* I
pET-30a(+)-His-pGSDMD-CT	CCGGAATTCATGGGGCCCGCGGAGGACCAG	*Eco*R I
	ACGCGTCGACCTAGCAGAGCTGGCTGAG	*Sal* I
pcDNA3.1(+)-pGSDMD-CT	CCGGAATTCATGGGGCCCGCGGAGGACCAG	*Eco*R I
	GCTCTAGACTAGCAGAGCTGGCTGAG	*Xba* I
pET-30a(+)-His-pGSDMD D279A	CTGAAGTTCCAGTCAGCCGGGCCCGCGGAGG	
	GACTTCAAGGTCAGTCGGCCCGGGCGCCTCC	

Restriction enzyme recognition sites are underlined in each sequence and listed in the right column.

**Table 2:** Plasmids used in experiments.

Name of plasmids	Details
pUC57-pcaspase-1	Stored in our laboratory
pUC57-pGSDMD	Stored in our laboratory
pET-30a(+)-pcaspase-1 p20	Prepared by this study, encoding 120-297aa of pcaspase-1 with a N-terminal 6×His tag
pET-30a(+)-His-pcaspase-1	Prepared by this study, encoding full-length pcaspase-1 with a N-terminal 6×His tag
pET-30a(+)-pcaspase-1-His	Prepared by this study, encoding full-length pcaspase-1 with a C-terminal 6×His tag
pET-30a(+)-Δpcaspase-1-His	Prepared by this study, encoding pcaspase-1 without the 1-120aa prodomain and with a C-terminal 6×His tag
pcDNA3.1(+)-pcaspase-1	Prepared by this study, encoding full-length pcaspase-1
pEGFP-C1-pGSDMD	Prepared by this study, encoding full-length pGSDMD with a N-terminal fused enhanced green fluorescent protein
pET-30a(+)-His-pGSDMD D279A	Prepared by this study, encoding a pGSDMD mutant of which the aspartate (D)279 is replaced with an alanine (A)
pET-30a(+)-His-pGSDMD-CT	Prepared by this study, encoding 280-489aa of pGSDMD with a N-terminal 6×His tag
pcDNA3.1(+)-pGSDMD-CT	Prepared by this study, encoding 280-489aa of pGSDMD
pET-30a(+)-His-pGSDMD-NT	Prepared by our laboratory, encoding 1-279aa of pGSDMD with a N-terminal 6×His tag
pcDNA3.1(+)-pGSDMD-NT	Prepared by our laboratory, encoding 1-279aa of pGSDMD
pET-30a(+)-His-pGSDMD	Prepared by our laboratory, encoding full-length pGSDMD with a N-terminal 6×His tag
pcDNA3.1(+)-pGSDMD	Prepared by our laboratory, encoding full-length pGSDMD

A mouse monoclonal antibody recognizing the C-terminal domain of pGSDMD was generated in our laboratory (the process for preparing the monoclonal antibody is described in detail in another manuscript currently under review). The mouse monoclonal antibody against the p20 subunit of pcaspase-1 was generated using the prokaryotically expressed and purified recombinant pcaspase-1p20. Anti-β-actin (2P2) mouse mAb (M20011,Abmart), mouse anti-His tagmAb (TA-02,ZSGB-BIO), horseradish peroxidase (HRP)-conjugated goat anti-mouse IgG (H+L) (ZB-2305, ZSGB-BIO), and HRP-conjugated goat anti-rabbit IgG (H+L) (ZB-2301, ZSGB-BIO) were commercially available.

### Piglet Tissue and Protein Extraction

The tissues of piglets, which had been orally challenged with ETEC (F5+), were provided by Dr. Ping Zhang, Northeast Agricultural University. The tissues were cut into small pieces and then frozen in liquid nitrogen. The frozen tissues were ground into powder. The powder was suspended in RIPA buffer (supplied with 1% PMSF) and placed on ice for 10 min. The mixtures were centrifuged at 12,000 × g for 15 min at 4°C, and the supernatant was collected and subjected to Western blot analysis.

### Cell Culture

Human embryonic kidney (HEK293) cells, intestinal porcine enterocytes (IPEC-J2), and Madin–Darby bovine kidney (MDBK) cells were cultured in Dulbecco’s Modified Eagle’s Medium (GIBCO, Shanghai, China) supplemented with 10% fetal bovine serum (FBS) (ABW, Shanghai, China). Mouse fibroblast cells (L929) were cultured in RPMI medium 1640 (GIBCO, Shanghai, China) containing 10% FBS. Cells were all incubated at 37°C in a 5% CO_2_ incubator.

### Transfection and Drug Treatment

For transfection, HEK293 cells were seeded into six-well cell culture plates at a density of 2×10^5^ cells. The cells were grown at 37°C overnight and then transfected with different plasmids, as indicated in the Results section, using a polyethylenimine linear transfection reagent (BIOHUB, Shanghai, China) according to the manufacturer’s instructions. For inhibition experiments, Z-VAD-FMK (A1902, APExBIO) or VX-765 (A8238, APExBIO) was added to the cells 4 h after transfection. The cells were harvested at the indicated time points and lysed in RIPA buffer (supplied with 1% PMSF) on ice for 10 min. The mixtures were centrifuged at 12,000 × g for 15 min at 4°C, and the supernatant was collected and subjected to Western blot analysis.

### Lactate Dehydrogenase Assay

HEK293, IPEC-J2, L929, and MDBK cells were seeded in 24-well plates at a density of 1.5×10^5^ cells/well. After an overnight growth, the cells were transfected with either pcDNA3.1(+)-pGSDMD (1 μg/well), pcDNA3.1(+)-pGSDMD-NT (1 μg/well), or pcDNA3.1(+)-pGSDMD-CT (1 μg/well). At the indicated time points after transfection, lactate dehydrogenase (LDH) activity in the culture supernatants was measured using an LDH Cytotoxicity Assay Kit (Beyotime, Beijing, China) following the manufacturer’s instruction.

### Microscope Observation

To determine the morphological change of transfected cells, cells were observed under an inverted microscope, and the static bright-field cell images were captured. Propidium iodide at 1 μg/mL (Beyotime, Beijing, China) was added concurrently for imaging experiments.

### Preparation of Recombinant pGSDMD (His-rpGSDMD) and His-rpGSDMD D279A

His-rpGSDMD was prokaryotically expressed and purified as described in a previous study (22). His-rpGSDMD D279A was prepared following the same protocol.

### Preparation of Activate rpcaspase-1

To obtain the active form of rpcaspase-1, pET-30a(+)-His-pcaspase-1, pET-30a(+)-pcaspase-1-His, and pET-30a(+)-Δpcaspase-1-His were transformed into *E. coli* Rosetta (DE3)™. Recombinant proteins were expressed by inducing with 0.2 mM IPTG at 25°C for 4 h and shaking at 220 r/min. The bacteria were harvested by centrifuging at 5,000 ×g at 4°C for 15 min and washed twice with ice-cold phosphate buffered solution (PBS). The bacteria were finally resuspended in PBS and lysed by sonication. The expression and autoprocessing of rpcaspase-1 in *E. coli* were analyzed by sodium dodecyl sulfate-polyacrylamide gel electrophoresis (SDS-PAGE) and visualized through Western blot analysis using mouse anti-rpcaspase-1 p20 mAb. The supernatant of bacteria lysate was collected after centrifugation at 12,000 ×g at 4°C for 15 min. The rpcaspase-1 activity was determined using Ac-YVAD-*p*NA (chromogenic substrate of caspase-1) (P9701,Beyotime). Briefly, 89 μL of the supernatant of bacteria lysate was mixed with 10 μL 10×reaction buffer (0.5 M HEPES [pH 7.5], 30 mM EDTA, 1.5 M NaCl, 0.05% [v/v] Tween 20, and 0.1 M DTT) and 1 μL Ac-YVAD-*p*NA solution (20 mM). The mixtures were incubated at 37°C for 7 h. Absorbance was measured at 405 nm at one-hour intervals. The experiment was performed in triplicate.

### *In Vitro* Cleavage Assay

First, 40 μL of purified His-rpGSDMD and His-rpGSDMD D279A (8 μg in total) was mixed, respectively, with different volumes of the supernatant of the *E. coli* lysate that showed caspase-1 activity and 10 μL 10× reaction buffer. PBS was added to the mixtures to a final volume of 100 μL. The mixtures were incubated at 37°C for different periods. The cleavage of His-rpGSDMD by His-rpcaspase-1 was analyzed by SDS-PAGE and visualized through Western blot analysis.

The ability of rpcaspase-3-His in cleaving His-rpGSDMD was also tested and analyzed by SDS-PAGE and Western blot assay.

Z-VAD-FMK (A1902, APExBIO), VX-765 (A8238, APExBIO), or Z-DEVD-FMK (caspase-3 inhibitor) (A1920,APExBIO) was used in several experiments to determine the specificity of His-rpcaspase-1 and rpcaspase-3-His in cleaving His-rpGSDMD.

### Bactericidal Assay

*E.coli* Rosetta (DE3)™ was transformed with pET-30a(+) expressing pGSDMD, pGSDMD-NT, or pGSDMD-CT. The transformants were cultured in LB medium containing 30 μg/mL kanamycin (Kan) at 37°C to OD_600nm_ that reached 0.6–0.8. Bacteria cells were diluted and grown on LB agar plates containing 30 μg/mL Kan and with or without 0.5 mM IPTG. After incubation at 37°C overnight, colony forming units on the plates were counted and statistically calculated.

In another independent experiment, *E. coli* transformants were cultured in LB medium containing 30 μg/mL Kan at 37°C to OD_600nm_ that reached 0.6–0.8. Next, IPTG was added into the medium. The cultures were further incubated at 37°C for 6 h. The OD_600nm_ value of the cultures were detected at one-hour intervals.

### Western Blot Assay

The protein concentration in samples was quantified using BCA protein assay kit (P0012,Beyotime). Proteins were then subjected to SDS-PAGE and transferred to nitrocellulose membranes. The membranes were blocked with 5% skim milk in PBS plus 0.05% (v/v) Tween 20 (PBST) at room temperature for 1 h. The membranes were then incubated with any of the mouse anti-rpGSDMD mAb (1:2,000), mouse anti-rpcaspase-1 p20 mAb, anti-β-actin (2P2) mouse mAb (1:5,000), and mouse anti-His tag mAb (1:5,000) at 4°C overnight and then incubated with HRP-conjugated goat anti-mouse IgG(H+L)(1:5,000) at room temperature for 1 h. Membranes were washed thrice with PBST for 5 min after each incubation step. Detection was conducted using BeyoECL Star (Beyotime, Beijing, China) following the manufacturer’s instructions.

### Statistical Analysis

Statistical analysis was conducted using GraphPad Prism 8 software, and all experiments were performed in triplicate. The two-sample student *t* test and one-way ANOVA was used for comparisons between groups. A *p* value<0.05 was considered statistically significant.

## Results

### Activation of pcaspase-1 and Cleavage of pGSDMD in ETEC (F5+) Challenged Piglet

**Figure 1:**
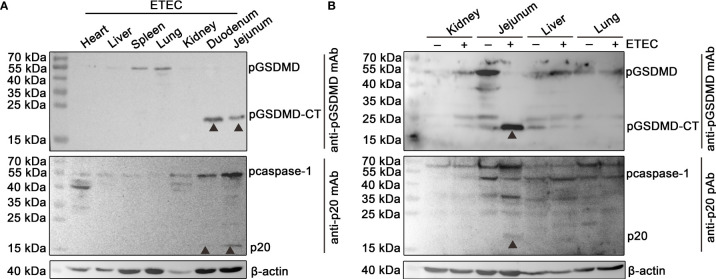
Determination of the activation of pcaspase-1 and cleavage of pGSDMD in tissues of the piglet. **(A)** Determination of the activation of pcaspase-1 and cleavage of pGSDMD in the tissues of ETEC (F5+) challenged piglet. The heart, liver, spleen, lung, kidney, duodenum and jejunum of a piglet orally challenged with 1×10^11^CFUof ETEC (F5+). **(B)** Difference in pcaspase-1 activation and pGSDMD cleavage in tissues between the healthy and ETEC (F5+) challenged piglets. The activation of pcaspase-1 and cleavage of pGSDMD in the kidneys, jejunums, livers and lungs of the piglets were analyzed by western blot assay. GSDMD: full-length GSDMD; GSDMD-CT: C-terminal cleavage product of GSDMD; p20: mature caspase-1.

The protein samples of the heart, liver, spleen, lung, kidney, duodenum, and jejunum of a piglet challenged with ETEC (F5+) were analyzed by Western blot assay. The results show that pcaspase-1 p20 and pGSDMD-CT can be detected in the duodenum and jejunum of the piglet but not in the other five organs (**Figure 1A**). The results also indicate that the activation of pcaspase-1 and the cleavage of pGSDMD in the piglet occurred simultaneously. The pcaspase-1 activation and pGSDMD cleavage occurred in the jejunum of the piglet challenged with ETEC (F5+) but not in the jejunum of the healthy piglet (**Figure 1B**). Considering that the pcaspase-1 activation and the pGSDMD cleavage are consistent in the tissue distribution, we speculate that pcaspase-1 activation and pGSDMD cleavage are closely associated.

### Preparation of Active rpcaspase-1

**Figure 2:**
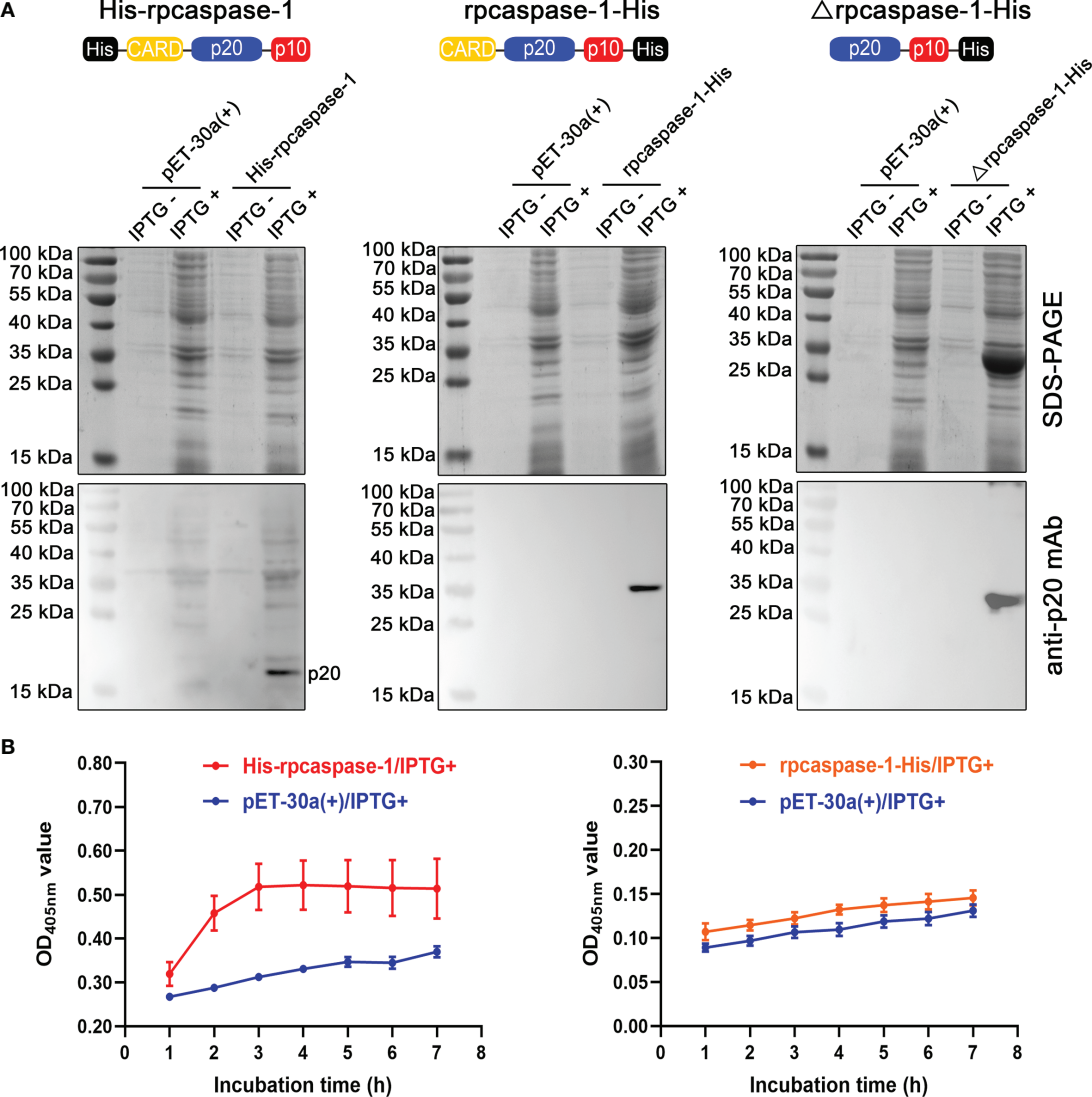
Expression and determination of enzymatic activity of recombinant pcaspase-1. **(A)** Determination of the expression of His-rpcaspase-1, rpcaspase-1-His and Δrpcaspase-1-His by SDS-PAGE and Western blot assays using anti-rpcaspase-1 p20 mAb. *E.coli* competent cells were transformed with either pET-30a(+), pET-30a(+)-His-pcaspase-1, pET-30a(+)-pcaspase-1-His or pET-30a(+)-Δpcaspase-1-His. IPTG was added into the liquid cultures of the transformants. The liquid cultures without IPTG were used as background controls. **(B)** The enzymatic activity of His-rpcaspase-1 and rpcaspase-1-His was determined by incubating the *E. coli* lysates containing the recombinant proteins with Ac-YVAD-*p*NA(a caspase-1substrate) and measuring the OD_405 nm_ values of the mixtures. Results shown are means from three technical replicates.

To obtain the active rpcaspase-1, three strategies were adopted (**Figure 2A**). The SDS-PAGE showed that only the Δrpcaspase-1-His was highly expressed in *E. coli*. By contrast, the expression of His-rpcaspase-1 and rpcaspase-1-His could not be determined by SDS-PAGE due to the poor expression efficiency (**Figure 2A**). Western blot assays were hence performed. The results show that p20 subunit can only be detected in *E. coli* that expressed the His-rpcaspase-1 by mouse anti-pcaspase-1 p20 monoclonal antibody (**Figure 2A**).

The enzymatic activity of rpcaspase-1 in pET-30a(+)-His-pcaspase-1 and pET-30a(+)-pcaspase-1-His transformed *E. coli* was then examined by using caspase-1 substrate (Ac-YVAD-*p*NA). The results show that His-rpcaspase-1 can cleave Ac-YVAD-*p*NA, but rpcaspase-1-His did not display any caspase activity (**Figure 2B**). The cleavage of Ac-YVAD-*p*NA by His-rpcaspase-1 reached its peak within 3 h, because the OD_405nm_ value of the reaction mixture did not increase after 3 h. The His-rpcaspase-1 was not purified for two reasons. First, the expression level of the His-rpcaspase-1 was extremely low. Second, the His-CARD region of the His-rpcaspase-1 was removed in the self-activation process.

### pGSDMD Is a Substrate of pcaspase-1

**Figure 3:**
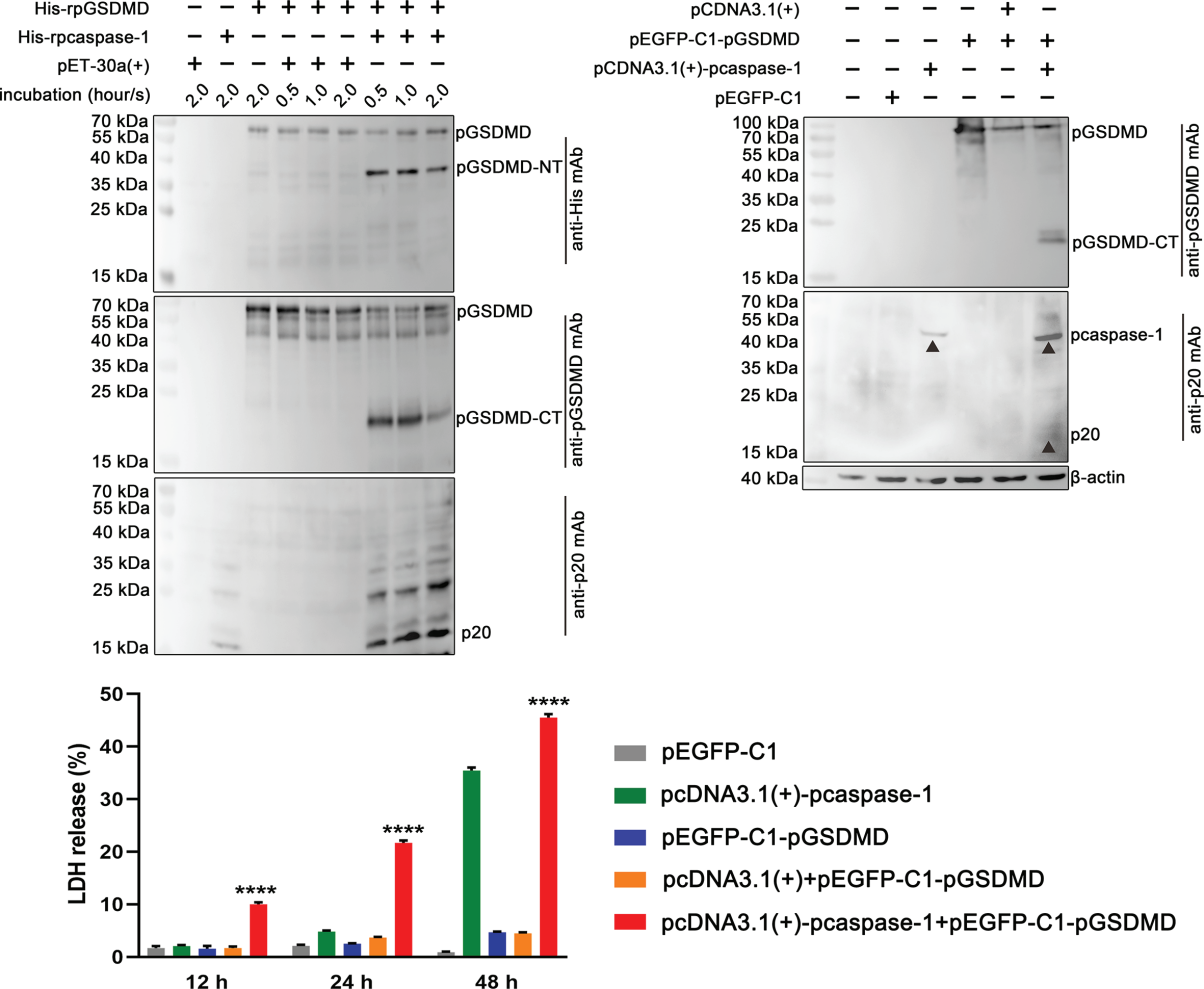
*In vitro* cleavage of pGSDMD by pcaspase-1. **(A)** Cleavage of His-rpGSDMD by His-rpcaspase-1 in a cell-free system. Purified His-rpGSDMD was incubated with *E. coli* lysate containing His-rpcaspase-1 for 0.5, 1 and 2 h. The products were analyzed by western blot assays using mouse Anti-His mAb, mouse anti-rpGSDMD mAb and mouse anti-rpcaspase-1 p20 mAb. The mixtures of His-rpGSDMD and the cell lysate of pET30a(+) transformed *E. coi l* were used as controls. **(B)** Cleavage of pGSDMD by pcaspase-1 in HEK293 cells. HEK293 cells were transfected pEGFP-C1-pGSDMD and pcDNA3.1(+)-pcaspase-1 simultaneously. The Cells transfected with either of the two plasmids and the cells co-transfected with pEGFP-C1-pGSDMD and pcDNA3.1(+) severed as controls. 48 h after the transfection, whole cell lysates were analyzed by mouse anti-rpGSDMD mAb. **(C)** Determination of the cellular LDH release of the transfected cells. The supernatant of the transfected HEK293 cells was collected 12, 24 and 48 h after the transfection and the LDH release of the cells was measured. Values are the means of triplicates and shown as means ± SD. ****p < 0.0001. GSDMD, full-length GSDMD; GSDMD-NT, N-terminal cleavage product of GSDMD; GSDMD-CT, C-terminal cleavage product of GSDMD; p20, mature caspase-1.

His-rpGSDMD was incubated with the supernatant of the *E. coli* lysate that contains active His-rpcaspase-1. The cleavage of His-rpGSDMD was determined by Western blot assays using anti-His tag monoclonal antibody and anti-pGSDMD monoclonal antibody as the primary antibodies. The results reveal that two proteins with molecular weights of approximately 40 kDa and 20 kDa can be recognized by anti-His tag monoclonal antibody and anti-pGSDMD monoclonal antibody, respectively (**Figure 3A**). The molecular weights of the two proteins are inconsistent with the predicated caspase-1 cleavage products of the His-rpGSDMD (a 38-kDa rpGSDMD-NT fragment involving a 7-kDa His-tag) and a 22-kDa rpGSDMD-CT fragment. The two protein fragments were not detected in mixtures of His-rpGSDMD and the supernatant of the *E. coli* lysate transformed with pET-30a(+) (**Figure 3A**).

To determine whether pcaspase-1 can cleave pGSDMD in eukaryocytes, HEK293 cells were co-transfected with pEGFP-C1-pGSDMD and pcDNA3.1(+)-pcaspase-1 for 48 h. A protein with a molecular weight similar to that of the predicted pGSDMD-CT was detected (**Figure 3B**). This protein was not detected in control cells and cells transfected with the plasmids, as indicated in **Figure 3B**. The results of LDH release assay show that the cells co-transfected with pcDNA3.1(+)-pcaspase-1 and pEGFP-C1-pGSDMD released more LDH than cells transfected with pcDNA3.1(+)-pcaspase-1 or pEGFP-C1-pGSDMD and control cells (**Figure 3C**).

These results indicate that pGSDMD may be a substrate of pcaspase-1 and that the cleavage products can cause cell membrane damage.

### Caspase Inhibitors Abolished the Cleavage of pGSDMD by pcaspase-1

**Figure 4:**
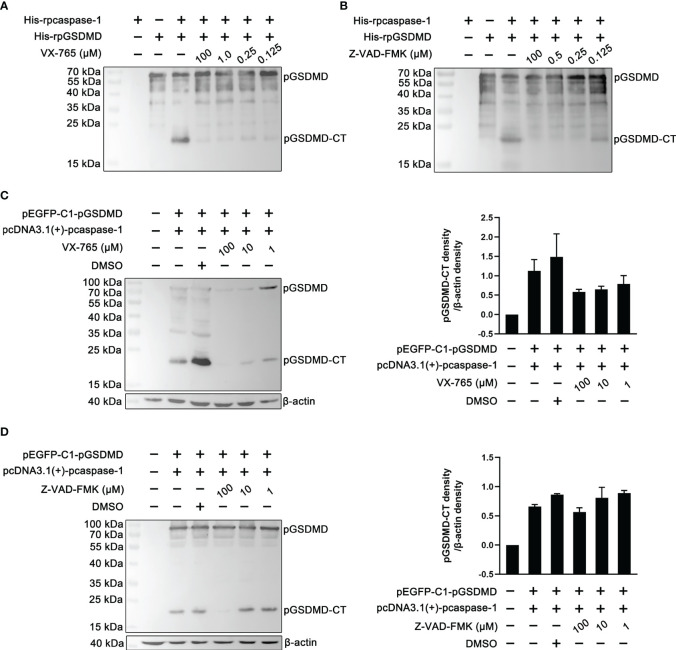
The effect of VX-765 and Z-VAD-FMK on the cleavage of pGSDMD by pcaspase-1. His-rpGSDMD was incubated with His-rpcaspase-1 in the presence of VX-765 **(A)** or Z-VAD-FMK **(B)**, the mixtures were analyzed by western blot analysis using mouse anti-rpGSDMD mAb. HEK293 cells were transfected with pEGFP-C1-pGSDMD and pcDNA3.1(+)-pcaspase-1 simultaneously, and then, VX-765 **(C)** or Z-VAD-FMK **(D)** were used to treat the cells for 18 h. The whole cell lysates were analyzed by mouse anti-rpGSDMD mAb based western blot assay(left panel). The density of pGSDMD-CT bands was quantified using the ImageJ software and normalized to β-actin (right panel).

To determine whether the enzymatic activity of the recombinantly expressed pcaspase-1 is necessary for pGSDMD cleavage, cleavage assays were performed in the presence of VX-765 (a caspase-1 inhibitor) and Z-VAD-FMK (a pan-caspase inhibitor). The results show that both VX-765 and Z-VAD-FMK inhibited the pGSDMD cleavage in the cell-free system (**Figures 4A, B**) and HEK293 cells in a dose-dependent manner (**Figures 4C, D**). The results indicate that the enzymatic activity of pcaspase-1 is required for pGSDMD cleavage.

### D279 Is Essential for pGSDMD Cleavage by pcaspase-1

**Figure 5:**
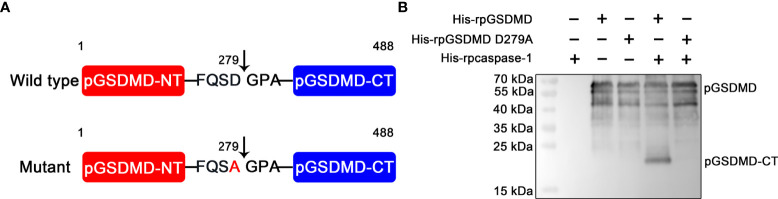
D279A mutation abolished the cleavage of His-rpGSDMD by His-rpcaspase-1. **(A)** The diagram of deduced cleavage site of pcaspase-1 in pGSDMD. The D279 was replaced with an alanine in the His-rpGSDMD D279A mutant. **(B)** His-rpGSDMD D279A and His-rpGSDMD were incubated with His-rpcaspase-1, respectively. The products were analyzed by Western blot analysis using mouse anti-rpGSDMD mAb.

On the basis of previous reports (8, 23), we speculated that the recognition motif of pcaspase-1 in pGSDMD is _276_Phenylalanine-Glutamine-Serine-Aspartic acid_279_ (_276_FQSD_279_). We then generated and prepared a mutant of pGSDMD (named His-rpGSDMD D279A), of which the D279 was replaced with an alanine (A) (**Figure 5A**). As expected, the D279A mutation completely abolished the cleavage of His-rpGSDMD by His-rpcaspase-1 (**Figure 5B**). The result confirms that the D279 in pGSDMD molecule is essential for its cleavage by pcaspase-1.

### pGSDMD-NT Causes Cell Membrane Damage

**Figure 6:**
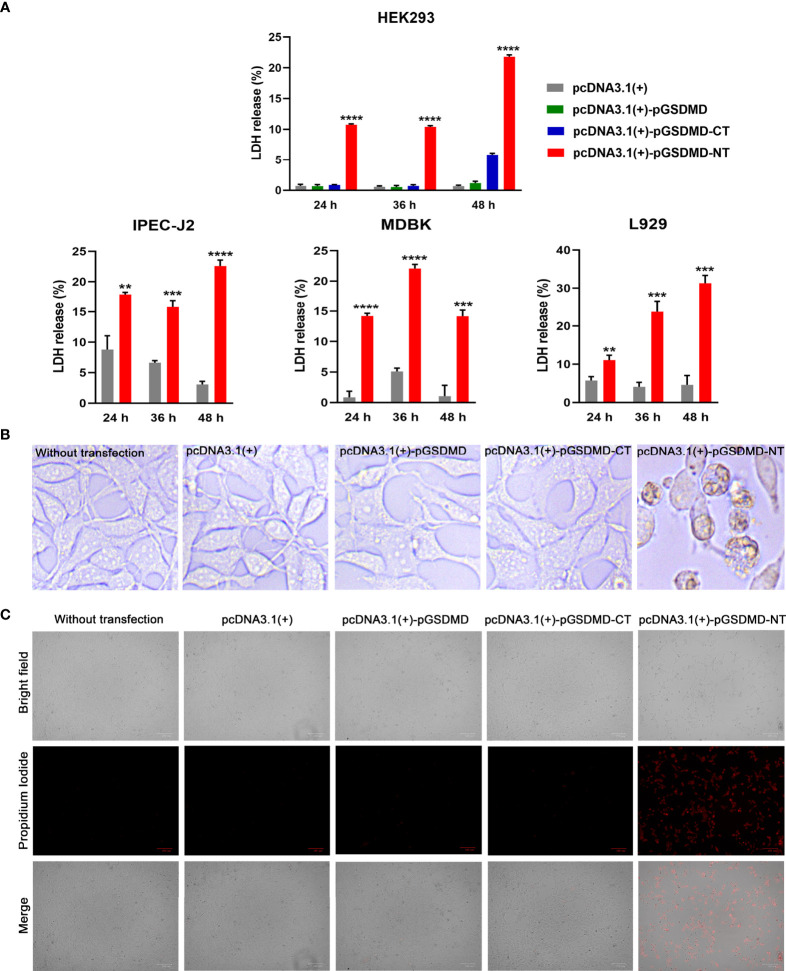
pGSDMD-NT caused cell membrane damage. **(A)** IPEC-J2, HEK293, L929 and MDBK cells were transfected with pcDNA3.1(+)-pGSDMD, pcDNA3.1(+)-pGSDMD-CT, pcDNA3.1(+)-pGSDMD-NT, or pcDNA3.1(+). The release of LDH was determined at 24, 36 and 48 h. Values are the means of triplicates and shown as means ± SD. **p < 0.01, ***p < 0.001, ****p < 0.0001. **(B)** The representative pictures of the HEK293 cells transfected with pcDNA3.1(+)-pGSDMD, pcDNA3.1(+)-pGSDMD-CT, pcDNA3.1(+)-pGSDMD-NT, or pcDNA3.1(+) at 24 h after the transfection. **(C)** The transfected HEK293 cells were stained with PI and observed under aninverted fluorescence microscope.

To determine the effect of pGSDMD-NT on the cell membrane’s integrity, pcDNA3.1(+)-pGSDMD-NT was transfected into IPEC-J2, HEK293, L929, and MDBK cells. The LDH release of the cells was measured. The results show that the cells transfected with pcDNA3.1(+)-pGSDMD-NT released more LDH than cells transfected with pcDNA3.1(+)-pGSDMD-CT, pcDNA3.1(+)-pGSDMD or pcDNA3.1(+) and control cells (**Figure 6A**).

In contrast to pGSDMD-CT and full-length pGSDMD, pGSDMD-NT could cause extensive cell death in HEK293 cells with apparent pyroptosis morphology (**Figure 6B**). The results of PI staining assays show that pcDNA3.1(+)-pGSDMD-NT transfected HEK293 cells uptake more PI than cells transfected with pcDNA3.1(+)-pGSDMD or pcDNA3.1(+) and control cells (**Figure 6C**).

The results indicate that pGSDMD-NT can destroy the cell membrane’s integrity.

### pGSDMD-NT Exhibits Bactericidal Activity

**Figure 7:**
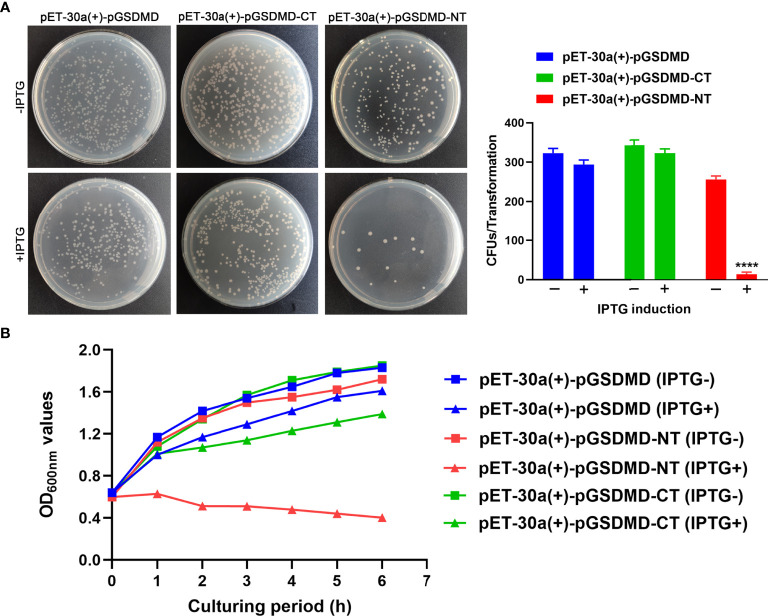
rpGSDMD-NT exhibits bactericidal activity. **(A)** *E. coli* that can express His-rpGSDMD, His-rpGSDMD-NT (1–279 residues) and His-rpGSDMD-CT (280–489 residues) grown on the plate s in the presence or absence of IPTG for 12 h. Left panel shows the representative pictures of the plates. The number of colonies on the plates were counted and statistically calculated (right panel). Values are the means of triplicates and shown as means ± SD. ****p < 0.0001. **(B)** The OD_600nm_ values of the liquid cultures of *E. coli* transformants expressing His-rpGSDMD, His-rpGSDMD-NT and His-rpGSDMD-CT.

The bactericidal activity of pGSDMD, pGSDMD-NT, and pGSDMD-CT was determined in the *E. coli* system. His-rpGSDMD, His-rpGSDMD-NT, and His-rpGSDMD-CT were expressed in *E. coli* cells with the IPTG induction. Colony counting (**Figure 7A**) and the measurement of OD_600nm_ value of the bacteria cultures (**Figure 7B**) show that the expression of His-rpGSDMD-NT, but not His-rpGSDMD and His-rpGSDMD-CT, in *E. coli* cells killed the bacteria efficiently.

### pcaspase-3 Can Cleave pGSDMD

**Figure 8:**
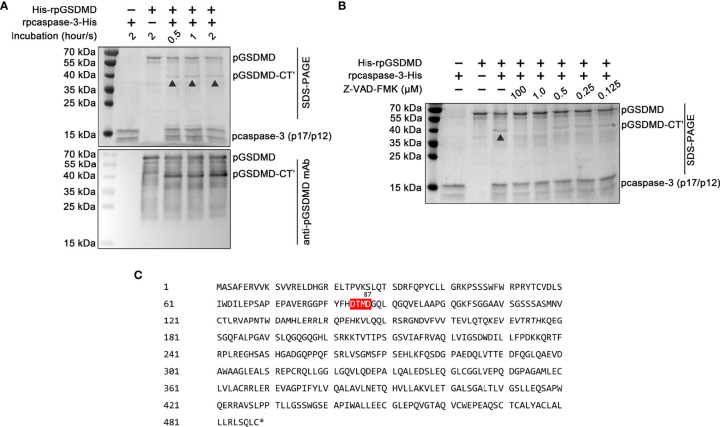
Cleavage of His-rpGSDMD by rpcaspase-3-His. **(A)** Purified His-rpGSDMD was incubated with purified rpcaspase-3-His for 0.5, 1 and 2 h. The cleavage products of His-rpGSDMD were analyzed by SDS-PAGE and western blot assay. **(B)** The effect of Z-DEVD-FMK on rpcaspase-3-His mediated His-rpGSDMD cleavage. **(C)** The diagram of deduced cleavage site of pcaspase-3 in pGSDMD.

Previous studies have shown that human and mice GSDMDs are the substrates of human caspase-3 and mouse caspase-3, respectively (24). Thus, we also examined whether pcaspase-3 can cleave pGSDMD. Our results showed that rpcaspase-3-His can indeed cleave His-rpGSDMD. In the mixture of rpcaspase-3-His and His-rpGSDMD, a protein with a molecular weight of approximately 40 kDa can be detected by SDS-PAGE and Western blot assay (**Figure 8A**). Considering that the anti-pGSDMD monoclonal antibody targets the pGSDMD-CT domain, our results indicate that the 40-kDa protein contains the pGSDMD-CT domain. In addition, the cleavage of His-rpGSDMD by rpcaspase-3-His in the cell-free system was inhibited by Z-DEVD-FMK (a caspase-3 inhibitor) in a dose-dependent manner (**Figure 8B**). This result confirms that the cleavage of His-rpGSDMD is indeed mediated by rpcaspase-3-His. By analyzing the pGSDMD’s primary structure, we found a motif that can be recognized by caspase-3, located at the 84aa to 87aa positions of the pGSDMD molecule (**Figure 8C**). The molecular weights of the two predicted cleavage products are approximately 8 kDa and 43 kDa, respectively. The predicted large cleavage product involves the pGSDMD-CT. The characteristics of the predicted large cleavage product are consistent with the 40-kDa protein observed on the gel and blot. Therefore, we speculate that the pcaspase-3 can cleave the pGSDMD between D87 and G88.

## Discussion

Although previous studies have shown that pGSDMD is cleaved when porcine cells are infected with different pathogens (19–21), how pGSDMD is cleaved and the effect of pGSDMD cleavage on the fate of cells remain unclear. A recent work showed that Seneca Valley Virus 3C protease can cleave pGSDMD after the glutamine 193 and glutamine 277 (23). As far as we know, the above study is a pilot research on the cleavage mechanism of pGSDMD. However, from the host’s perspective, the underlying mechanism of pGSDMD cleavage remains unknown.

In the present study, we first determined that the pcaspase-1 activation and the pGSDMD cleavage occurred in the small intestinal tissue of a piglet challenged with ETEC (F5+) (**Figure 1**). To our best knowledge, the present work is the first investigation that uses a pGSDMD-specific monoclonal antibody as a research tool. A previous study showed that CSFV infection caused the pcaspase-1 activation and the pGSDMD cleavage in peripheral lymphoid organs of infected pigs (19). These results indicated that the pcaspase-1 activation and the pGSDMD cleavage generally have a similar tissue distribution pattern in pigs. Considering that GSDMD is mainly cleaved by caspase-1 in humans and mice (8, 25), we speculated that pcaspase-1 activation and pGSDMD cleavage are closely related.

To determine the ability of pcaspase-1 to cleave pGSDMD in the cell-free system, we prepared active rpcaspase-1 in a prokaryotic expression system. Previous reports presented two strategies to obtain active human and mouse caspase-1 in the *E. coli* expression system. The first one is to purify the recombinantly expressed caspase-1 p20 and p10 subunits in *E. coli* cells under denaturing condition and then dilute the mixture of p10 and p20 to allow them to refold and assemble into active rcaspase-1 (26). We have tried this strategy, but pcaspase-1 p10 failed to express in *E. coli* cells. The other strategy is to express the full-length rcaspase-1 in *E. coli* cells. Active rcaspase-1 can be obtained after the self-activation process (27). In the current study, His-rpcaspase-1, rpcaspase-1-His, and Δrpcaspase-1-His were expressed in *E. coli* (**Figure 2**). The results showed that active rpcaspase-1 was only obtained from the His-rpcaspase-1 transformant. In addition, the fused-His tag at the amino-terminal domain of His-rpcaspase-1 was removed along with the prodomain during the self-activation process (the recombinant protein cannot be recognized by anti-His tag antibody; data not shown). A previous study demonstrated that the recombinantly expressed mouse caspase-1 with carboxyl-terminal fused His-tag and with or without prodomain could be autoprocessed into p10 and p20 subunits (27). Silkworm (*Bombyxmori*) caspase-1 and oyster (*Crassostreagigas*) caspase-1 can also be autoprocessed in the prokaryotic expression system (28). Our results showed that rpcaspase-1-His could not be fully autoprocessed and that Δrpcaspase-1-His was expressed as an inclusion body. Although removal of the prodomain in rpcaspase-1-His was observed (the protein in the rpcaspase-1-His sample that can be recognized by the anti-pcaspase-1 antibodies has the same molecular weight as Δrpcaspase-1-His), the separation of p20 and p10 subunits failed (**Figure 2**). The results indicated that a carboxyl-terminal fused oligopeptide significantly affected the auto processing of rpcaspase-1. For the above reasons, the *E. coli* lysate expressing the His-rpcaspase-1 was used as the source of active rpcaspase-1 without purification. The cleavage of His-rpGSDMD by His-rpcaspase-1 could therefore be determined by Western blot assay but not SDS-PAGE.

Previous studies have shown that GSDMD is a substrate of caspase-1 in humans and mice (8, 29). In the current study, we found that pcaspase-1 is capable of cleaving pGSDMD in the cell-free system and HEK293 cells (**Figure 3**). In addition, the caspase inhibitors can abolish the pGSDMD cleavage by pcaspase-1 (**Figure 4**), indicating that the enzymatic activity of pcaspase-1 is critical to its ability to cleave pGSDMD. These results reveal the relationship between the pcaspase-1 activation and pGSDMD cleavage that we observed in the intestinal tissue of the ETEC-challenged piglet (**Figure 1**).

The caspase-1 cleavage sites in human and murine GSDMDs are _272_FLTD|G_276_ and _273_LLSD|G_277_, respectively (8). A previous work speculated that pGSDMD 1-279aa is the N-terminal fragment of pGSDMD caused by caspase-1 cleavage (23). Analysis of the pGSDMD’s primary structure showed that the amino acid composition around the 279aa position of the pGSDMD molecule is _276_FQSD|G_280_ (**Figure 5A**). This motif is similar to the caspase-1 cleavage sites of human and murine GSDMDs. Considering that caspases cleave proteins and peptide C-terminal to aspartic acid residues (30), D279 in the _276_FQSD|G_280_ motif of the pGSDMD molecule was mutated to an alanine to verify the role of this motif in the pGSDMD cleavage by pcaspase-1 in the current study. The cleavage assay in cell-free system showed that the D279A mutation completely abolished the pGSDMD cleavage by pcaspase-1 (**Figure 5B**). This result indicates that D279 is essential for the pcaspase-1-mediated pGSDMD cleavage and the FQSD|G is a new amino acid composition pattern that can be recognized and cleaved by caspase-1.

The GSDMD cleavage caused the disassociation of GSDMD-NT and GSDMD-CT (8, 31, 32). Human and murine GSDMD-NT can punch holes in the cell membrane and trigger pyroptosis (33, 34). The current study found that the pGSDMD-NT can cause damage to the cell membrane. Overexpression of pGSDMD-NT led to increased LDH release and PI entry in the cells (**Figure 6**). However, the immune staining result of a previous study showed that the overexpressed pGSDMD 1-277aa mainly aggregates into spots in the cells but is not located on the cell membrane (23). The exact mechanism of pGSDMD-NT to destroy the cell membrane’s integrity is therefore worthy of further study. The pGSDMD-NT killed the *E. coli* Rosetta (DE3)™ rapidly when the former was expressed in the bacteria cells (**Figure 7**). This result does not exceed the expectation, because human and murine GSDMD-NT (33, 35) and pGSDMD 1-277aa (23) exhibited bactericidal activity in such system. However, our result regarding the lack of bactericidal activity of full-length pGSDMD (**Figure 7**) is inconsistent with the result of the previous study (23). This difference may be attributed to the different *E. coli* competent cells used in the two studies or the difference in the recombinant protein induction conditions.

Notably, pcaspase-3 was able to cleave pGSDMD *in vitro*, which is consistent with previous studies in humans and mice (24, 36). This result indicates that pcaspase-3 may also play roles in regulating pGSDMD-mediated pyroptosis.

In conclusion, our study shows that pGSDMD can be cleaved by pcaspase-1 and the NT domain of pGSDMD can cause damage in cell membrane and lead to pyroptosis-like cell death. Our study provides significant information about the mechanism of pyroptosis in porcine cells.

## Data Availability Statement

The original contributions presented in the study are included in the article/supplementary material. Further inquiries can be directed to the corresponding authors.

## Author Contributions

WZ designed the experiments, analyzed the results, supervised the work, and drafted the manuscript. YS performed most of the experiments and drafted the manuscript. JS prepared the mouse anti-porcine GSDMD monoclonal antibody. MW prepared active pcaspase-3 protein. BM and JW supervised the work. All authors read and approved the final version of the manuscript.

## Funding

This work was supported by the University Nursing Program for Young Scholars with Creative Talents in Heilongjiang Province, grant number UNPYSCT-2017019 and “Academic Backbone” Project of Northeast Agricultural University, grant number 17XG09.

## Conflict of Interest

The authors declare that the research was conducted in the absence of any commercial or financial relationships that could be construed as a potential conflict of interest.

## Publisher’s Note

All claims expressed in this article are solely those of the authors and do not necessarily represent those of their affiliated organizations, or those of the publisher, the editors and the reviewers. Any product that may be evaluated in this article, or claim that may be made by its manufacturer, is not guaranteed or endorsed by the publisher.
